# 2,4,6-Trinitro-*N*-[4-(phenyl­diazen­yl)phen­yl]aniline

**DOI:** 10.1107/S160053681101004X

**Published:** 2011-03-23

**Authors:** Graham Smith, Urs D. Wermuth

**Affiliations:** a Faculty of Science and Technology, Queensland University of Technology, GPO Box 2434, Brisbane, Queensland 4001, Australia

## Abstract

The title compound, C_18_H_12_N_6_O_6_, was prepared from the reaction of 4-(phenyl­diazen­yl)aniline (aniline yellow) with picryl­sulfonic acid. The dihedral angle formed by the two benzene rings of the diphenyl­diazenyl ring system is 6.55 (13)° and that formed by the rings of the picrate–aniline ring system is 48.76 (12)°. The mol­ecule contains an intra­molecular aniline–nitro N—H⋯O hydrogen bond.

## Related literature

For the reaction of picryl chloride with isomeric amino­benzoic acids, see: Crocker & Matthews (1911 ▶). For the application of the title compound in dyeing technology, see: Beretta (1926 ▶);. For structural data on *N*-picryl-substituted anilines, see: Forlani *et al.* (1992 ▶); Pan *et al.* (2007 ▶); Smith *et al.* (2007) ▶; Braun *et al.* (2008 ▶); Smith *et al.* (2009 ▶). For diazenyl-protonated salts of aniline yellow, see: Mahmoudkhani & Langer (2001 ▶); Smith *et al.* (2009 ▶, 2011 ▶).
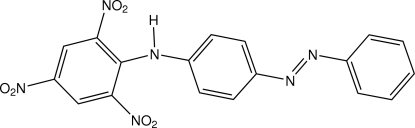


## Experimental

### 

#### Crystal data

C_18_H_12_N_6_O_6_
*M* *_r_* = 408.34
Monoclinic, 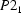

*a* = 7.4255 (4) Å
*b* = 7.6613 (4) Å
*c* = 16.1510 (9) Å
β = 98.160 (5)°
*V* = 909.51 (9) Å^3^
*Z* = 2
Mo *K*α radiation
μ = 0.12 mm^−1^
*T* = 200 K
0.30 × 0.30 × 0.15 mm

#### Data collection

Oxford Diffraction Gemini-S CCD-detector diffractometer
Absorption correction: multi-scan (*CrysAlis PRO*; Oxford Diffraction, 2010 ▶) *T*_min_ = 0.920, *T*_max_ = 0.990
6768 measured reflections
2297 independent reflections
1407 reflections with *I* > 2σ(*I*)
*R*_int_ = 0.036

#### Refinement

*R*[*F* ^2^ > 2σ(*F* ^2^)] = 0.037
*wR*(*F* ^2^) = 0.058
*S* = 0.86
2297 reflections
271 parameters
1 restraint
H-atom parameters not refined
Δρ_max_ = 0.15 e Å^−3^
Δρ_min_ = −0.14 e Å^−3^

### 

Data collection: *CrysAlis PRO* (Oxford Diffraction, 2010 ▶); cell refinement: *CrysAlis PRO*; data reduction: *CrysAlis PRO*; program(s) used to solve structure: *SIR92* (Altomare *et al.*, 1994 ▶); program(s) used to refine structure: *SHELXL97* (Sheldrick, 2008 ▶) within *WinGX* (Farrugia, 1999 ▶); molecular graphics: *PLATON* (Spek, 2009 ▶); software used to prepare material for publication: *PLATON*.

## Supplementary Material

Crystal structure: contains datablocks global, I. DOI: 10.1107/S160053681101004X/lh5220sup1.cif

Structure factors: contains datablocks I. DOI: 10.1107/S160053681101004X/lh5220Isup2.hkl

Additional supplementary materials:  crystallographic information; 3D view; checkCIF report

## Figures and Tables

**Table 1:** Hydrogen-bond geometry (Å, °)

*D*—H⋯*A*	*D*—H	H⋯*A*	*D*⋯*A*	*D*—H⋯*A*
N1—H1⋯O21*A*	0.86	1.98	2.607 (3)	129

**Fig. 1.**
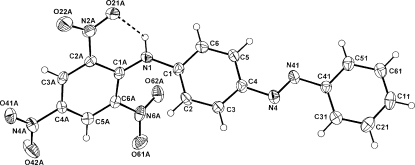
The molecular structure of the title compound. The intramolecular hydrogen bond is shown as a dashed line and displacement ellipsoids are drawn at the 40% probability level.

## supplementary crystallographic information

### Comment

The diazo-dye precursor aniline yellow [4-(phenyldiazenyl)aniline] reacts with strong acids to form purple-black to red-black diazenyl-protonated salts (Mahmoudkhani & Langer, 2001; Smith *et al.*, 2009, 2011). However, our 1:1 stoichiometric reaction of aniline yellow with 2,4,6-trinitrobenzenesulfonic acid (picrylsulfonic acid) in 50% ethanol-water atypically gave orange-red crystals. This indicated a substitution reaction typical of this acid with anilines, giving *N*-picryl products with elimination of the sulfonate group. Reaction of picryl chloride with the isomeric aniline carboxylates to give similar products was reported by Crocker & Matthews (1911) while the application of picryl substituted azoanilines including the title compound, (I), in dyeing, was discussed by Beretta (1926). A number of structures of picryl-substituted anilines and other aromatic amines have been reported (*e.g.* Forlani *et al.*, 1992; Braun *et al.*, 2008; Pan *et al.*, 2007; Smith *et al.*, 2007).

In the title compound, (I), picryl-substitution of the aniline group of the 4-(phenyldiazenyl)aniline molecule has occurred. The molecular structure of (I) is shown Fig. 1. The diphenyldiazenyl ring system is non-planar [torsion angles C3—C4—N4—N41 and C51—C41—N41—N4: 175.4 (2) and -169.5 (2)°, respectively] as is the picrate to aniline ring system [torsion angles C2A—C1A—N1—C1 and C6—C1—N1—C1A: 152.5 (2) and 156.3 (3)° respectively]. Within the picrate moiety, one of the two *ortho*-related nitro groups is rotated out of the benzene plane [torsion angle C5A—C6A—N6A—O62A, 147.1 (2)°], while the other, which is associated with an intramolecular hydrogen bond [N1—H···O21A (Table 1)] is close to coplanar [C1A—C2A—N2A—O22A, -173.3 (2)°]. The *para*-related nitro group is also essentially coplanar with the ring [C3A—C4A—N4A—O42A, 172.0 (2)°].  There is one short intermolecular non-bonding nitro group interaction [O41A···O42A^i^, 2.860 (3) Å: symmetry code (i) -*x* + 1, *y* - 1/2, -*z*]. In addition, there are weak π···π ring interactions with a centroid to centroid distance 3.7744 (16) Å.

### Experimental

The title compound was synthesized by heating together under reflux for 10 minutes, 1 mmol quantities of 4-(phenyldiazenyl)aniline (aniline yellow) and 2,4,6-trinitrobenzenesulfonic acid (picrylsulfonic acid) in 50 ml of 50% ethanol-water. After concentration to *ca* 30 ml, partial room temperature evaporation of the hot-filtered solution gave orange-red prisms of (I) from which a specimen was cleaved for the X-ray analysis.

### Refinement

All H-atoms were included in the refinement in calculated positions and were allowed to ride with C—H = 0.93 Å or N—H = 0.86 Å and with *U*_iso_(H) = 1.2*U*_eq_(C, N). In the absence of significant anomalous dispersion effects Friedel pairs were merged for the final cycles of refinement.

### Crystal data

**Table:**

C_18_H_12_N_6_O_6_	*F*(000) = 420
*M**_r_* = 408.34	*D*_x_ = 1.491 Mg m^−^^3^
Monoclinic, *P*2_1_	Mo *K*α radiation, λ = 0.71073 Å
Hall symbol: P 2yb	Cell parameters from 2105 reflections
*a* = 7.4255 (4) Å	θ = 3.5–28.5°
*b* = 7.6613 (4) Å	µ = 0.12 mm^−^^1^
*c* = 16.1510 (9) Å	*T* = 200 K
β = 98.160 (5)°	Plate, orange-red
*V* = 909.51 (9) Å^3^	0.30 × 0.30 × 0.15 mm
*Z* = 2	

### Data collection

**Table:**

Oxford Diffraction Gemini-S CCD-detector diffractometer	2297 independent reflections
Radiation source: Enhance (Mo) X-ray source	1407 reflections with *I* > 2σ(*I*)
graphite	*R*_int_ = 0.036
Detector resolution: 16.077 pixels mm^-1^	θ_max_ = 28.5°, θ_min_ = 3.5°
ω scans	*h* = −9→9
Absorption correction: multi-scan (*CrysAlis PRO*; Oxford Diffraction, 2010)	*k* = −10→10
*T*_min_ = 0.920, *T*_max_ = 0.990	*l* = −21→21
6768 measured reflections	

### Refinement

**Table:**

Refinement on *F*^2^	Primary atom site location: structure-invariant direct methods
Least-squares matrix: full	Secondary atom site location: difference Fourier map
*R*[*F*^2^ > 2σ(*F*^2^)] = 0.037	Hydrogen site location: inferred from neighbouring sites
*wR*(*F*^2^) = 0.058	H-atom parameters not refined
*S* = 0.86	*w* = 1/[σ^2^(*F*_o_^2^) + (0.021*P*)^2^] where *P* = (*F*_o_^2^ + 2*F*_c_^2^)/3
2297 reflections	(Δ/σ)_max_ < 0.001
271 parameters	Δρ_max_ = 0.15 e Å^−^^3^
1 restraint	Δρ_min_ = −0.14 e Å^−^^3^

### Special details

**Table:**

Geometry. Bond distances, angles *etc*. have been calculated using the rounded fractional coordinates. All su's are estimated from the variances of the (full) variance-covariance matrix. The cell e.s.d.'s are taken into account in the estimation of distances, angles and torsion angles
Refinement. Refinement of *F*^2^ against ALL reflections. The weighted *R*-factor *wR* and goodness of fit *S* are based on *F*^2^, conventional *R*-factors *R* are based on *F*, with *F* set to zero for negative *F*^2^. The threshold expression of *F*^2^ > σ(*F*^2^) is used only for calculating *R*-factors(gt) *etc*. and is not relevant to the choice of reflections for refinement. *R*-factors based on *F*^2^ are statistically about twice as large as those based on *F*, and *R*- factors based on ALL data will be even larger.

### Fractional atomic coordinates and isotropic or equivalent isotropic displacement parameters (Å^2^)

**Table:**

	*x*	*y*	*z*	*U*_iso_*/*U*_eq_	
O21A	0.7894 (3)	0.0405 (2)	0.33042 (15)	0.0557 (8)	
O22A	0.8395 (3)	−0.0131 (3)	0.20499 (15)	0.0673 (10)	
O41A	0.7046 (3)	0.3737 (3)	−0.01934 (12)	0.0524 (8)	
O42A	0.5573 (3)	0.6113 (3)	−0.00428 (13)	0.0733 (10)	
O61A	0.4815 (3)	0.8150 (3)	0.26498 (14)	0.0582 (9)	
O62A	0.4238 (2)	0.6124 (3)	0.35215 (12)	0.0467 (7)	
N1	0.6941 (3)	0.3543 (3)	0.36913 (14)	0.0379 (8)	
N2A	0.7860 (3)	0.0827 (3)	0.25675 (17)	0.0438 (10)	
N4	0.8515 (3)	0.7986 (3)	0.64591 (15)	0.0376 (8)	
N4A	0.6334 (3)	0.4775 (3)	0.02299 (15)	0.0403 (9)	
N6A	0.4931 (3)	0.6655 (3)	0.29240 (16)	0.0397 (9)	
N41	0.7964 (3)	0.7460 (3)	0.71112 (15)	0.0388 (8)	
C1	0.7256 (3)	0.4761 (4)	0.43607 (17)	0.0343 (10)	
C1A	0.6660 (3)	0.3836 (3)	0.28559 (17)	0.0304 (9)	
C2	0.8086 (3)	0.6339 (3)	0.42983 (17)	0.0367 (10)	
C2A	0.7143 (3)	0.2547 (3)	0.22900 (18)	0.0323 (9)	
C3	0.8466 (3)	0.7381 (4)	0.50001 (17)	0.0363 (10)	
C3A	0.7034 (3)	0.2855 (3)	0.14441 (17)	0.0325 (10)	
C4	0.8030 (3)	0.6843 (4)	0.57641 (18)	0.0357 (10)	
C4A	0.6397 (3)	0.4442 (4)	0.11242 (17)	0.0304 (9)	
C5	0.7209 (4)	0.5221 (4)	0.58222 (18)	0.0420 (11)	
C5A	0.5793 (3)	0.5688 (3)	0.16261 (17)	0.0321 (10)	
C6	0.6810 (4)	0.4184 (4)	0.51302 (18)	0.0429 (11)	
C6A	0.5887 (3)	0.5373 (3)	0.24652 (17)	0.0301 (9)	
C11	0.9043 (4)	1.0521 (4)	0.92620 (19)	0.0475 (11)	
C21	0.9363 (3)	1.1240 (4)	0.8506 (2)	0.0455 (11)	
C31	0.9046 (3)	1.0274 (4)	0.77855 (17)	0.0361 (10)	
C41	0.8401 (3)	0.8580 (3)	0.78142 (17)	0.0313 (10)	
C51	0.8078 (3)	0.7876 (4)	0.85652 (18)	0.0391 (10)	
C61	0.8423 (4)	0.8851 (4)	0.92962 (19)	0.0437 (11)	
H1	0.69280	0.24650	0.38380	0.0450*	
H2	0.83930	0.67080	0.37880	0.0440*	
H3	0.90220	0.84590	0.49580	0.0430*	
H3A	0.73870	0.19990	0.10920	0.0390*	
H5	0.69290	0.48390	0.63360	0.0500*	
H5A	0.53250	0.67380	0.14000	0.0390*	
H6	0.62490	0.31080	0.51700	0.0510*	
H11	0.92550	1.11840	0.97480	0.0570*	
H21	0.97920	1.23780	0.84890	0.0540*	
H31	0.92630	1.07520	0.72800	0.0430*	
H51	0.76280	0.67450	0.85820	0.0470*	
H61	0.82320	0.83690	0.98050	0.0520*	

### Atomic displacement parameters (Å^2^)

**Table:**

	*U*^11^	*U*^22^	*U*^33^	*U*^12^	*U*^13^	*U*^23^
O21A	0.0839 (16)	0.0368 (12)	0.0427 (14)	−0.0079 (11)	−0.0033 (12)	0.0064 (11)
O22A	0.0965 (18)	0.0423 (14)	0.0679 (18)	0.0221 (13)	0.0283 (14)	−0.0003 (12)
O41A	0.0661 (14)	0.0584 (14)	0.0361 (14)	−0.0002 (12)	0.0191 (11)	−0.0091 (11)
O42A	0.0923 (18)	0.0854 (17)	0.0449 (15)	0.0435 (16)	0.0196 (13)	0.0222 (14)
O61A	0.0700 (16)	0.0370 (14)	0.0701 (18)	0.0057 (11)	0.0187 (12)	−0.0105 (11)
O62A	0.0385 (11)	0.0683 (14)	0.0349 (13)	−0.0036 (11)	0.0107 (10)	−0.0101 (11)
N1	0.0475 (14)	0.0359 (13)	0.0305 (15)	−0.0133 (12)	0.0065 (12)	0.0030 (12)
N2A	0.0492 (16)	0.0339 (16)	0.0470 (19)	−0.0023 (12)	0.0022 (14)	0.0000 (14)
N4	0.0358 (13)	0.0478 (15)	0.0286 (16)	−0.0031 (12)	0.0022 (11)	−0.0014 (12)
N4A	0.0354 (14)	0.0543 (17)	0.0318 (16)	0.0028 (13)	0.0067 (12)	0.0020 (14)
N6A	0.0276 (13)	0.0496 (18)	0.0414 (17)	−0.0032 (12)	0.0031 (12)	−0.0160 (14)
N41	0.0429 (14)	0.0470 (15)	0.0268 (15)	−0.0013 (13)	0.0058 (11)	−0.0008 (13)
C1	0.0327 (16)	0.0389 (18)	0.0303 (18)	−0.0076 (14)	0.0014 (13)	−0.0048 (15)
C1A	0.0253 (15)	0.0353 (16)	0.0302 (18)	−0.0072 (13)	0.0028 (13)	−0.0062 (14)
C2	0.0347 (15)	0.0505 (19)	0.0246 (17)	−0.0113 (14)	0.0031 (12)	0.0061 (15)
C2A	0.0312 (15)	0.0283 (15)	0.0365 (18)	−0.0030 (13)	0.0019 (13)	−0.0032 (14)
C3	0.0359 (15)	0.0401 (17)	0.0318 (19)	−0.0123 (14)	0.0012 (13)	−0.0004 (15)
C3A	0.0278 (15)	0.0359 (17)	0.0343 (18)	−0.0073 (13)	0.0057 (13)	−0.0111 (14)
C4	0.0337 (16)	0.0433 (18)	0.0280 (18)	−0.0039 (14)	−0.0027 (13)	0.0004 (15)
C4A	0.0273 (14)	0.0397 (17)	0.0244 (16)	−0.0027 (13)	0.0039 (12)	−0.0024 (14)
C5	0.0560 (19)	0.0454 (19)	0.0251 (18)	−0.0117 (16)	0.0073 (14)	0.0015 (14)
C5A	0.0227 (14)	0.0357 (17)	0.0370 (19)	−0.0007 (13)	0.0010 (13)	0.0007 (14)
C6	0.0527 (18)	0.0401 (18)	0.0359 (19)	−0.0138 (14)	0.0065 (15)	0.0029 (14)
C6A	0.0229 (13)	0.0359 (16)	0.0323 (18)	−0.0024 (13)	0.0063 (12)	−0.0076 (14)
C11	0.0380 (17)	0.063 (2)	0.040 (2)	0.0134 (17)	0.0005 (15)	−0.0199 (17)
C21	0.0331 (17)	0.0445 (18)	0.057 (2)	−0.0013 (15)	0.0001 (15)	−0.0113 (17)
C31	0.0280 (15)	0.0444 (18)	0.035 (2)	0.0026 (14)	0.0014 (14)	0.0023 (15)
C41	0.0304 (15)	0.0346 (17)	0.0277 (18)	0.0030 (14)	−0.0004 (13)	−0.0009 (14)
C51	0.0387 (16)	0.0432 (19)	0.0345 (19)	0.0078 (14)	0.0018 (14)	−0.0046 (15)
C61	0.0434 (17)	0.053 (2)	0.0338 (19)	0.0094 (16)	0.0025 (14)	−0.0003 (17)

### Geometric parameters (Å, °)

**Table:**

O21A—N2A	1.230 (4)	C3A—C4A	1.378 (4)
O22A—N2A	1.221 (3)	C4—C5	1.393 (4)
O41A—N4A	1.217 (3)	C4A—C5A	1.369 (4)
O42A—N4A	1.222 (3)	C5—C6	1.369 (4)
O61A—N6A	1.227 (3)	C5A—C6A	1.369 (4)
O62A—N6A	1.226 (3)	C11—C61	1.364 (4)
N1—C1	1.422 (4)	C11—C21	1.390 (4)
N1—C1A	1.354 (4)	C21—C31	1.371 (4)
N2A—C2A	1.467 (3)	C31—C41	1.386 (4)
N4—N41	1.250 (3)	C41—C51	1.379 (4)
N4—C4	1.429 (4)	C51—C61	1.390 (4)
N4A—C4A	1.461 (4)	C2—H2	0.9300
N6A—C6A	1.472 (3)	C3—H3	0.9300
N41—C41	1.423 (3)	C3A—H3A	0.9300
N1—H1	0.8600	C5—H5	0.9300
C1—C6	1.402 (4)	C5A—H5A	0.9300
C1—C2	1.367 (4)	C6—H6	0.9300
C1A—C2A	1.426 (4)	C11—H11	0.9300
C1A—C6A	1.418 (3)	C21—H21	0.9300
C2—C3	1.382 (4)	C31—H31	0.9300
C2A—C3A	1.378 (4)	C51—H51	0.9300
C3—C4	1.382 (4)	C61—H61	0.9300
			
C1—N1—C1A	129.4 (2)	C1—C6—C5	119.3 (3)
O21A—N2A—O22A	122.7 (2)	N6A—C6A—C1A	121.6 (2)
O21A—N2A—C2A	119.2 (2)	N6A—C6A—C5A	114.9 (2)
O22A—N2A—C2A	118.0 (3)	C1A—C6A—C5A	123.2 (2)
N41—N4—C4	112.9 (2)	C21—C11—C61	120.5 (3)
O41A—N4A—O42A	124.1 (2)	C11—C21—C31	120.1 (3)
O41A—N4A—C4A	119.1 (2)	C21—C31—C41	119.7 (3)
O42A—N4A—C4A	116.8 (2)	N41—C41—C51	114.7 (2)
O61A—N6A—O62A	125.3 (2)	N41—C41—C31	125.3 (2)
O61A—N6A—C6A	117.1 (2)	C31—C41—C51	120.0 (3)
O62A—N6A—C6A	117.5 (2)	C41—C51—C61	120.2 (3)
N4—N41—C41	114.4 (2)	C11—C61—C51	119.5 (3)
C1—N1—H1	115.00	C1—C2—H2	120.00
C1A—N1—H1	115.00	C3—C2—H2	120.00
N1—C1—C2	123.5 (2)	C2—C3—H3	120.00
C2—C1—C6	120.6 (3)	C4—C3—H3	120.00
N1—C1—C6	115.7 (3)	C2A—C3A—H3A	120.00
C2A—C1A—C6A	114.4 (2)	C4A—C3A—H3A	120.00
N1—C1A—C6A	125.2 (2)	C4—C5—H5	120.00
N1—C1A—C2A	120.4 (2)	C6—C5—H5	120.00
C1—C2—C3	119.5 (2)	C4A—C5A—H5A	120.00
C1A—C2A—C3A	122.2 (2)	C6A—C5A—H5A	120.00
N2A—C2A—C1A	122.7 (2)	C1—C6—H6	120.00
N2A—C2A—C3A	115.1 (2)	C5—C6—H6	120.00
C2—C3—C4	120.9 (3)	C21—C11—H11	120.00
C2A—C3A—C4A	119.5 (2)	C61—C11—H11	120.00
N4—C4—C5	123.9 (3)	C11—C21—H21	120.00
N4—C4—C3	117.0 (3)	C31—C21—H21	120.00
C3—C4—C5	119.1 (3)	C21—C31—H31	120.00
N4A—C4A—C5A	119.8 (2)	C41—C31—H31	120.00
N4A—C4A—C3A	119.1 (2)	C41—C51—H51	120.00
C3A—C4A—C5A	121.1 (3)	C61—C51—H51	120.00
C4—C5—C6	120.6 (3)	C11—C61—H61	120.00
C4A—C5A—C6A	119.3 (2)	C51—C61—H61	120.00
			
C1A—N1—C1—C2	−29.4 (4)	C6A—C1A—C2A—C3A	6.3 (3)
C1A—N1—C1—C6	156.3 (3)	N1—C1A—C6A—N6A	−14.0 (4)
C1—N1—C1A—C2A	152.5 (2)	N1—C1A—C6A—C5A	173.3 (2)
C1—N1—C1A—C6A	−27.7 (4)	C2A—C1A—C6A—N6A	165.7 (2)
O21A—N2A—C2A—C1A	7.0 (4)	C2A—C1A—C6A—C5A	−7.0 (3)
O21A—N2A—C2A—C3A	−175.4 (2)	C1—C2—C3—C4	0.5 (4)
O22A—N2A—C2A—C1A	−173.3 (2)	N2A—C2A—C3A—C4A	−179.3 (2)
O22A—N2A—C2A—C3A	4.3 (3)	C1A—C2A—C3A—C4A	−1.6 (4)
C4—N4—N41—C41	−179.2 (2)	C2—C3—C4—N4	178.5 (2)
N41—N4—C4—C3	175.4 (2)	C2—C3—C4—C5	0.5 (4)
N41—N4—C4—C5	−6.7 (4)	C2A—C3A—C4A—N4A	178.3 (2)
O41A—N4A—C4A—C3A	−8.9 (4)	C2A—C3A—C4A—C5A	−3.0 (4)
O41A—N4A—C4A—C5A	172.4 (2)	N4—C4—C5—C6	−178.9 (3)
O42A—N4A—C4A—C3A	172.0 (2)	C3—C4—C5—C6	−1.1 (4)
O42A—N4A—C4A—C5A	−6.7 (3)	N4A—C4A—C5A—C6A	−178.9 (2)
O61A—N6A—C6A—C1A	157.5 (2)	C3A—C4A—C5A—C6A	2.4 (4)
O61A—N6A—C6A—C5A	−29.3 (3)	C4—C5—C6—C1	0.8 (4)
O62A—N6A—C6A—C1A	−26.1 (3)	C4A—C5A—C6A—N6A	−170.2 (2)
O62A—N6A—C6A—C5A	147.1 (2)	C4A—C5A—C6A—C1A	2.9 (4)
N4—N41—C41—C31	12.6 (4)	C61—C11—C21—C31	0.4 (4)
N4—N41—C41—C51	−169.5 (2)	C21—C11—C61—C51	−1.2 (4)
N1—C1—C2—C3	−174.9 (2)	C11—C21—C31—C41	0.2 (4)
C6—C1—C2—C3	−0.8 (4)	C21—C31—C41—N41	178.0 (2)
N1—C1—C6—C5	174.7 (3)	C21—C31—C41—C51	0.1 (4)
C2—C1—C6—C5	0.2 (4)	N41—C41—C51—C61	−179.0 (2)
N1—C1A—C2A—N2A	3.5 (3)	C31—C41—C51—C61	−1.0 (4)
N1—C1A—C2A—C3A	−174.0 (2)	C41—C51—C61—C11	1.5 (4)
C6A—C1A—C2A—N2A	−176.3 (2)		

### Hydrogen-bond geometry (Å, °)

**Table:**

*D*—H···*A*	*D*—H	H···*A*	*D*···*A*	*D*—H···*A*
N1—H1···O21A	0.86	1.98	2.607 (3)	129
C3A—H3A···O22A	0.93	2.30	2.633 (3)	100
C5—H5···O61A^i^	0.93	2.58	3.453 (4)	157
C11—H11···O41A^ii^	0.93	2.56	3.068 (4)	115
C21—H21···O22A^iii^	0.93	2.56	3.425 (4)	155

Symmetry codes: (i) −*x*+1, *y*−1/2, −*z*+1; (ii) *x*, *y*+1, *z*+1; (iii) −*x*+2, *y*+3/2, −*z*+1.
